# Association and impact of hypertension defined using the 2017 AHA/ACC guidelines on the risk of atrial fibrillation in The Atherosclerosis Risk in Communities study

**DOI:** 10.1186/s12872-019-1259-0

**Published:** 2019-11-26

**Authors:** Anas Rattani, J’Neka S. Claxton, Mohammed K. Ali, Lin Y. Chen, Elsayed Z. Soliman, Alonso Alvaro

**Affiliations:** 1 Hubert Department of Global Health, Rollins School of Public Health, Emory University, Atlanta, GA USA; 2 Department of Epidemiology, Rollins School of Public Health, Emory University, 1518 Clifton Rd, CNR 3051, Atlanta, GA 30307 USA; 3 Department of Family and Preventive Medicine, School of Medicine, Emory University, Atlanta, GA USA; 4 Cardiovascular Division, Department of Medicine, University of Minnesota Medical School, Minneapolis, MN USA; 5 Epidemiological Cardiology Research Center, Department of Epidemiology and Prevention, Wake Forest School of Medicine, Winston-Salem, NC USA

**Keywords:** Atrial fibrillation, Hypertension, JNC7, ACC/AHA, Atherosclerosis risk in communities study

## Abstract

**Background:**

Hypertension is an established risk factor for the development of atrial fibrillation (AF). We evaluated the association and population impact of hypertension, defined using the new 2017 guidelines, on risk of AF.

**Methods:**

In this analysis, we included 14,915 participants in the Atherosclerosis Risk in Communities study without history of AF. Participants underwent blood pressure measurements at baseline and their antihypertensive medication use was assessed. Incident AF was ascertained from study electrocardiograms, hospital records and death certificates. Cox proportional models were used to estimate hazard ratios (HR) and 95% confidence intervals (CI) of AF among individuals with hypertension based on the JNC7 and 2017 ACC/AHA guidelines. Poisson models were used to obtain risk ratios and calculate population-attributable fractions (PAFs).

**Results:**

We identified 2891 cases of incident AF during 21.4 years of mean follow-up. Prevalence of hypertension was 34 and 48% under the JNC7 and 2017 ACC/AHA definitions, respectively. HRs (95%CI) of AF in hypertensives versus non-hypertensives were 1.44 (1.32, 1.56) and 1.37 (1.26, 1.48) after multivariable adjustment under the old and new guidelines, respectively. The corresponding PAF (95%CI) using the old and new guidelines were 11% (8, 13%) and 13% (9, 16%), respectively.

**Conclusions:**

Overall, our analysis shows that even though the prevalence of hypertension using the new criteria is 40% higher than with the old criteria, this does not translate into meaningful increases in AF attributable to hypertension. These results suggest that prevention or treatment of hypertension based on the new (versus old) guidelines may have limited impact on AF incidence.

## Background

Atrial fibrillation (AF) is a common chronic arrhythmia, affecting between 2.7–6.1 million people in the United States [1]. Common risk factors for AF include obesity, diabetes, smoking, heavy drinking, and hypertension [2]. Among the risk factors listed, hypertension has the largest population attributable fraction for AF incidence and plays a major role in the management and prognosis of AF [3–5]. Individuals with hypertension have a 1.7-fold higher risk of developing AF, with one in six cases of AF possibly due to hypertension [3]. Hypertension is very common among individuals with AF, with studies showing prevalence of 69 to 90% of hypertension among AF patients [4, 6, 7]. Thus, early detection and management of hypertension is key to preventing and managing AF.

The 7th Joint National Committee (JNC7) defined hypertension as a systolic blood pressure (SBP) ≥140 mmHg or diastolic blood pressure (DBP) ≥90 mmHg, regardless of age. Blood pressure was divided into the following categories: Normal: SBP < 120 and DBP < 80, Prehypertension: SBP 120–139 or DBP 80–89, Stage 1 hypertension: SBP 140–159 or DBP 90–99, and Stage 2 hypertension: SBP > 160 or DBP ≥ 100 [8, 9]. At the end of 2017, the American Heart Association/American College of Cardiology (AHA/ACC) released new guidelines lowering the threshold to define elevated blood pressure. The new recommended blood pressure diagnostic categories are: Normal: SBP < 120 and DBP < 80, Elevated: SBP 120–129 and DBP < 80, Stage 1 hypertension: SBP 130–139 or DBP 80–89, Stage 2 hypertension: SBP ≥140 or DBP ≥90 [10, 11]. This change means more individuals will be diagnosed with hypertension. For example, an analysis of NHANES data comparing the 2014 and 2017 hypertension guidelines reported an increase in the prevalence of hypertension from 32 to 45% [12]. However, it is uncertain whether individuals labeled as being hypertensive with the new guidelines are at similarly increased risk of AF, or whether the population impact of newly defined hypertension will have a similar impact in the incidence of AF.

The goal of this study was to evaluate the association between hypertension and risk of AF and the population attributable fraction of hypertension in AF using the new diagnostic categories in a prospective cohort free of cardiovascular disease and diabetes at baseline. Results from these analyses will contribute to inform the ideal blood pressure range for the prevention of AF as well as the potential population impact of preventing and treating hypertension under the new guidelines.

## Methods

### Study population

For the present analysis, we used data from the Atherosclerosis Risk in Communities (ARIC) study cohort. The ARIC cohort aims to investigate the epidemiology of atherosclerosis, clinical atherosclerotic diseases, and variation in cardiovascular risk factors, treatment, and disease. The cohort study began in 1987, recruiting participants from four U.S. communities: Washington County in Maryland, Forsyth County in North Carolina, city of Jackson in Mississippi, and the northwest suburbs of Minneapolis in Minnesota. There were approximately 4000 participants recruited from each community through probability sampling. The study enrolled 15,792 participants aged 45–64 (7082 were men and 11,526 were white). Detailed clinical, social, and demographic data were obtained at baseline in 1987–89. Participants have had additional evaluations in 1990–92, 1993–95, 1996–98, 2011–2013, and 2016–2017. The participants were also followed-up annually (biannually since 2012) by telephone to stay in contact, ascertain cardiovascular events, and to measure the health status of the cohort. More information about the design and objectives of the study can be found on the ARIC website as well as in published articles [13].

**Fig. 1:**
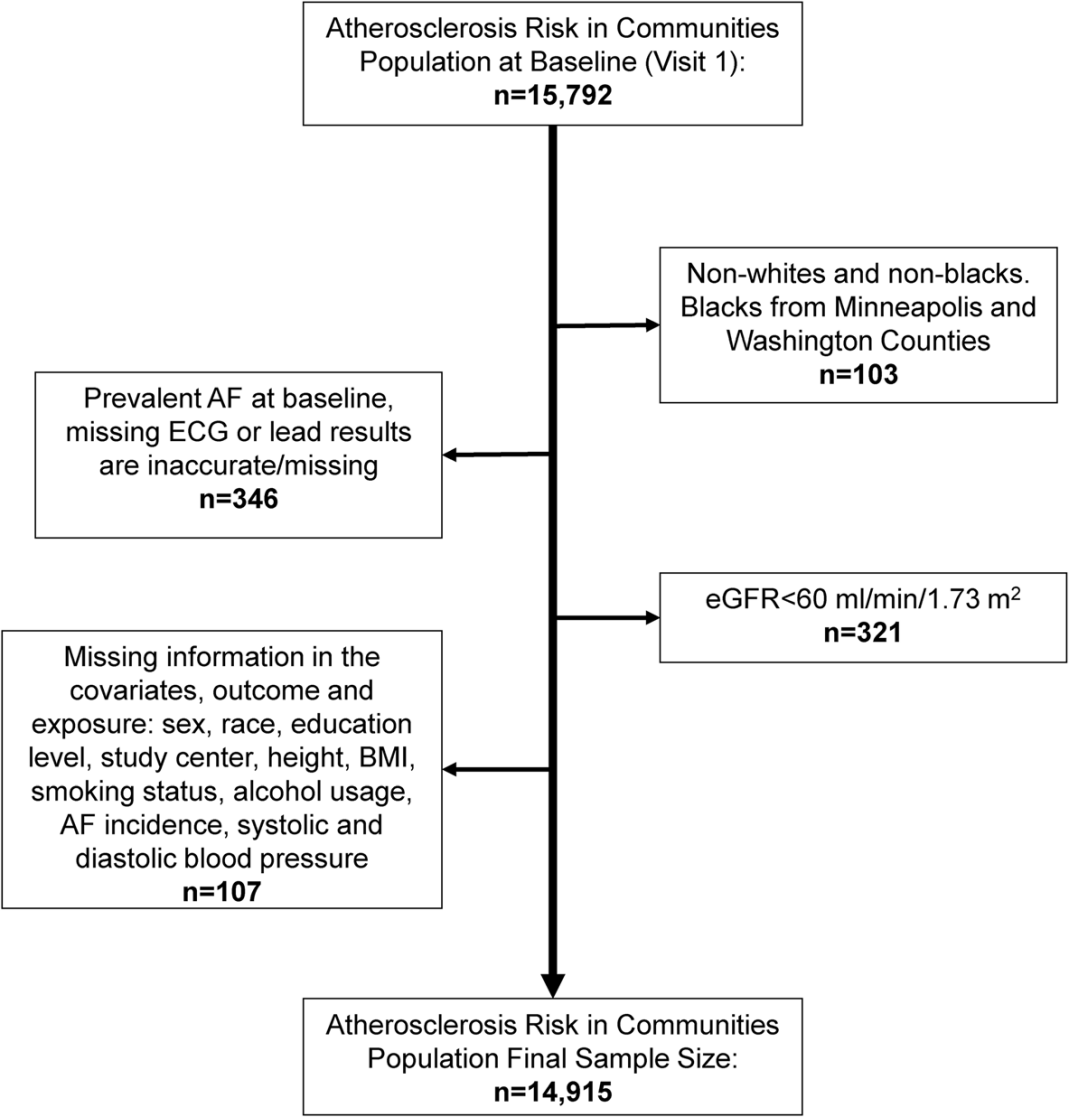
Flow Chart of Patients Excluded at Baseline, Atherosclerosis Risk in Communities Study, 1987–1989

For the present analysis, we included ARIC participants who had baseline blood pressure readings at visit 1 (1987–89). We excluded participants who had AF at baseline or missing ECG (*N* = 346), individuals of a race other than white or black, as well as blacks from the Minneapolis and Washington County Centers due to small numbers in those groups (*N* = 103), an eGFR value of less than 60 ml/min/1.73 m^2^ (*N* = 321), and participants who had missing values for the outcome, exposure, or covariates (*N* = 107). After excluding participants who did not meet our study criteria, our final sample size was 14,915 participants (Fig. 1 presents a flow chart for the final sample size).

### Assessment of blood pressure

At the baseline visit, sitting systolic and diastolic blood pressure was measured with a random zero sphygmomanometer 3 times at baseline after a 5-min rest. The second and third measurements were averaged and used in the analysis. Use of blood pressure lowering medications was ascertained by asking participants to bring to the visit all medications they had been using over the previous 2 weeks. Baseline blood pressure and use of antihypertensive medication was used to define hypertension categories.

### Assessment of incident AF

We used three methods to identify cases of AF in the ARIC cohort: ECG performed at study visits, hospital discharge codes, and death certificates. The ECG studies were performed with a 12-lead ECG during study exams. The data obtained was transmitted electronically to the ARIC Central ECG Reading Center and processed using the GE Marquette 12-SL program. Presence of AF in the ECG was identified by a computer algorithm and then confirmed by a cardiologist. A cardiologist also read over ECGs with any other rhythm abnormalities to reduce the possibility of any missed AF incidents. Hospitalizations during the study period were identified with follow-up phone calls and monitoring local hospitals. Information such as discharge codes were collected from these hospitals by abstractors. If a participant had discharge codes ICD-9-CM codes 427.31 or 427.32 (ICD-10-CM code I48.x after October 1, 2015), then they were considered to have AF. Cases where a participant had open heart surgery in association with AF were excluded. Finally, if a patient had codes such as ICD-9427.3 or ICD-10 I48 in their death certificates, then the participant was considered to have AF [14].

### Assessment of covariates

Sex, race, education (categorized as grade school, high school but no degree, high school graduate, vocational school, college or graduate/professional school), smoking status (categorized as never, former or current smoker), and alcohol usage (categorized as never, former, current drinker) were obtained via self-report, while height and weight were measured with participants wearing light clothing. We calculated body mass index (BMI) as weight in kilograms divided by height in meters squared. Diabetes was defined as a fasting blood glucose ≥126 mg/dL, non-fasting blood glucose ≥200 mg/dL, use of antidiabetic medication, or self-reported physician diagnosis of diabetes. Baseline stroke, coronary heart disease and heart failure were defined based on self-reported information.

### Statistical analysis

Analysis was conducted using SAS 9.4 statistical software (SAS Institute, Cary, NC). Cox proportional models were used to estimate the hazard ratios (HR) and 95% confidence interval (CI) of AF incidence among individuals with hypertension based on the JNC7 and 2017 AHA/ACC guidelines. For our independent variable, both the new and old hypertensive guidelines were divided into categories established by JNC7 and 2017 AHA/ACC, as indicated in Table 1. Participants using antihypertensive medication were labeled as having hypertension (JNC7) or stage 2 hypertension (2017 AHA/ACC) regardless of their visit blood pressure.

**Table 1:** Categories of Blood Pressure Established by JNC7 and 2017 ACC/AHA Guidelines

JNC 7				2017 ACC/AHA			
	SBP		DBP		SBP		DBP
Normal	< 120	and	< 80	Normal	< 120	and	< 80
Prehypertension	120–139	or	80–90	Elevated	120–129	and	< 80
Hypertension	≥140	or	≥90	Stage 1	130–139	or	80–89
				Stage 2	≥140	or	≥90

SBP/DBP measurements are in mmHg

*JNC 7* 7th Joint National Committee, *ACC/ AHA* American College of Cardiology/American Heart Association, *SBP* systolic blood pressure, *DBP* diastolic blood pressure

Two separate analyses were conducted to fully characterize the impact of changing the definition of hypertension. The first analysis considered hypertension as a binary variable. For the JNC7 guideline, prehypertension and normal were combined in the reference group. For the 2017 AHA/ACC guideline, elevated and normal blood pressure were combined as the reference group whereas stage 1 and stage 2 were combined to define hypertension. We conducted a stratified analysis by sex and race to explore effect modification. The second analysis considered hypertension classified into more specific categories in both the JNC7 and 2017 AHA/ACC guidelines. Using a normal blood pressure as the reference in both guidelines, we calculated HRs of AF among individuals with prehypertension/elevated, hypertension, stage 1, or stage 2. Covariate adjustment was done through two separate models. Model 1 adjusted for age, sex, and race, while model 2 additionally adjusted for education, study center, height, BMI, smoking status, alcohol use, diabetes, heart failure, coronary heart disease, and stroke.

We calculated population-attributable fractions (PAFs) of AF by hypertension categories to determine the possible impact of preventing hypertension on AF occurrence. PAFs were computed according to the following formula: PAF = pd_i_[(RR_i_-1)/RR_i_], where pd_i_ is the proportion of cases falling into *i*th exposure level and RR_i_ is the relative risk (RR) comparing *i*th exposure level with unexposed group (i = 0) [15]. Poisson models were used to estimate RRs. The offset in the Poisson model was calculated as the natural logarithm of the time from visit 1 to AF incidence, death or lost to follow up until December 31, 2015, whichever came first. Ninety five percent confidence intervals (95%CI) for the PAF were obtained applying the corresponding 95%CI of the RR to the PAF formula above.

## Results

### Basic demographic characteristics of participants in ARIC study

We included 14,915 eligible adults in our final sample. The mean age at baseline was 54.1 years old (SD = 5.7). The sample was 74% white and 55% women. The percentage of individuals taking hypertension medication in the cohort was 24%. Based on the JNC7 guidelines, 42% individuals had normal blood pressure, 24% of individuals were prehypertensive and 34% of individuals were hypertensive (Table 2). Based on the 2017 ACC/AHA guidelines, 42% individuals had normal blood pressure, 10% of individuals had elevated blood pressure, 14% of individuals were stage 1 hypertensive, and 34% of individuals were stage 2 hypertensive (Table 2).

**Table 2:** Demographic Characteristics of Participants in the ARIC Study Based on the JNC 7 and ACC/AHA Guideline Categories

JNC7 Categories	Normal	Prehypertension		Hypertension
N (%)	6249 (41.9)	3619 (24.3)		5047 (33.8)
Age (years)	52.8 (5.5)	54.5 (5.7)		55.5 (5.6)
White	5393 (86.3)	2750 (76.0)		2962 (58.7)
Women	3628 (58.1)	1779 (49.2)		2809 (55.7)
Completed high school	5200 (83.2)	2800 (77.4)		3454 (68.4)
Current smokers	1794 (28.7)	883 (24.4)		1232 (24.4)
Current drinkers	3869 (61.9)	2095 (57.9)		2466 (48.9)
BMI (kg/m^2^)	26.1 (4.3)	27.8 (5.2)		29.5 (5.9)
Height (cm)	168 (9)	169 (9)		168 (9)
SBP (mmHg)	106 (8)	126 (6)		135 (20)
DBP (mmHg)	66 (7)	76 (8)		81 (12)
Hypertension medication	–	–		3648 (72.3)
Diabetes	368 (5.9)	334 (9.2)		994 (19.7)
Heart failure	92 (1.5)	57 (1.6)		506 (10.0)
Coronary artery disease	216 (3.5)	130 (3.6)		351 (7.0)
Stroke	88 (1.4)	42 (1.2)		131 (2.6)
ACC/AHA categories	Normal	Elevated	Stage 1 hypertension	Stage 2 hypertension
N (%)	6249 (41.9)	1562 (10.5)	2057 (13.8)	5047 (33.8)
Age (years)	52.8 (5.5)	55.3 (5.7)	53.9 (5.7)	55.5 (5.6)
White	5393 (86.3)	1293 (82.8)	1457 (70.8)	2962 (58.7)
Women	3628 (58.1)	817 (52.3)	962 (46.8)	2809 (55.7)
Completed high school	5200 (83.2)	1206 (77.2)	1594 (77.5)	3454 (68.4)
Current smokers	1794 (28.7)	414 (26.5)	469 (22.8)	1232 (24.4)
Current drinkers	3869 (61.9)	916 (58.6)	1179 (57.3)	2466 (48.9)
BMI (kg/m^2^)	26.1 (4.3)	27.5 (5.0)	28.1 (5.3)	29.5 (5.9)
Height (cm)	168 (9)	169 (10)	170 (9)	168 (9)
SBP	106 (8)	124 (3)	128 (8)	135 (20)
DBP	66 (7)	71 (6)	80 (7)	81 (12)
Hypertension medication	–	–	–	3648 (72.3)
Diabetes	368 (5.9)	147 (9.4)	187 (9.1)	994 (19.7)
Heart failure	92 (1.5)	26 (1.7)	31 (1.5)	506 (10.0)
Coronary artery disease	216 (3.5)	64 (4.1)	66 (3.2)	351 (7.0)
Stroke	88 (1.4)	18 (1.2)	24 (1.2)	131 (2.6)

Numbers correspond to mean (SD) and N (percentages)

*JNC 7* 7th Joint National Committee, *ACC/ AHA* American College of Cardiology/American Heart Association, *SBP* systolic blood pressure, *DBP* diastolic blood pressure

### Association of hypertension (binary) with AF incidence using JNC 7 and 2017 ACC/AHA definitions

During a mean follow-up of 21.4 years, we identified 2891 cases of incident AF overall. Using the JNC7 definition, the incidence rate of AF per 1000 person-years were 7.5 and 12.5 for no hypertension and hypertension respectively. The HR of AF in hypertension compared to no hypertension was 1.70 (95% CI 1.57, 1.83) after adjusting for age, sex and race, and 1.44 (95% CI 1.32, 1.56) after multivariable adjustment. Corresponding AF rates using the 2017 AHA/ACC guidelines definition were 7.3 and 11.2 per 1000 person-years for no hypertension and hypertension, respectively. The HR of AF was 1.55 (95% CI 1.43, 1.67) after adjusting for age, sex and race and 1.37 (95% CI 1.26, 1.48) after multivariable adjustment (Table 3).

**Table 3:** Hazard Ratios (95% Confidence intervals) of Atrial Fibrillation According to Hypertension Defined According to JNC 7 and 2017 ACC/AHA Guidelines, ARIC 1987–2015

JNC 7	No hypertension	Hypertension
No. of AF cases	1665	1226
No. of participants	9868	5047
Person-years	220,591	98,426
Incidence rate (per 1000 PY)	7.5	12.5
Model 1 [HR (95%CI)]	1 (ref.)	1.70 (1.57, 1.83)
Model 2 [HR (95%CI)]	1 (ref.)	1.44 (1.32, 1.56)
2017 ACC/AHA	No hypertension	Hypertension
No. of AF cases	1283	1608
No. of participants	7811	7104
Person-years	175,925	143,092
Incidence rate (per 1000 PY)	7.3	11.2
Model 1 [HR (95%CI)]	1 (ref.)	1.55 (1.43, 1.67)
Model 2 [HR (95%CI)]	1 (ref.)	1.37 (1.26, 1.48)

Model 1: Adjusted for age, sex, and race

Model 2: Adjusted for age, sex, race, height, education, field center, body mass index, smoking, drinking status, diabetes, heart failure, coronary heart disease, and stroke

*JNC 7*: 7th Joint National Committee, *ACC/ AHA* American College of Cardiology/American Heart Association, *PY* person-years

In analyses stratified by sex and race, association of hypertension, as defined by both JNC7 and 2017 AHA/ACC guidelines, were similar across groups, with the exception of a significant interaction by sex in the 2017 AHA/ACC guidelines (*p* = 0.01) (Additional file 1: Table S1). Hypertension was more strongly associated with AF incidence in women (HR 1.55, 95% CI 1.38, 1.75) than in men (HR 1.23, 95% CI 1.10, 1.37).

### Association of blood pressure with AF using JNC 7 and 2017 ACC/AHA guideline categories

Using the JNC7 categories, the AF incidence rates per 1000 person-years were 6.6, 9.3, and 12.5 for normal blood pressure, prehypertension, and hypertension, respectively. The HRs (95% CI) of AF for prehypertension and hypertension, compared to normal blood pressure, were 1.24 (1.12, 1.36) and 1.58 (1.44, 1.74) respectively after multivariable adjustment. Using 2017 AHA/ACC guideline categories, the incidence rates for AF per 1000 person-years were 6.6, 10.3, 8.6, and 12.5 for normal, elevated, stage 1, and stage 2, respectively. The HR (95% CI) for elevated, stage 1 and stage 2, compared to normal blood pressure, were: 1.26 (1.11, 1.43), 1.21 (1.07, 1.37), and 1.58 (1.44, 1.74), respectively, after multivariable adjustment (Table 4).

**Table 4:** Hazard Ratios (95% Confidence Intervals) of Atrial Fibrillation by Categories of Blood Pressure According to JNC 7 and 2017 ACC/AHA Definitions, ARIC 1987–2015

JNC 7	Normal	Prehypertension		Hypertension
No. of AF cases	941	724		1226
No. of participants	6249	3619		5047
Person-years	142,742	77,849		98,426
Incidence rate (per 1000 PY)	6.6	9.3		12.5
Model 1 [HR (95%CI)]	1 (ref.)	1.30 (1.17, 1.43)		1.90 (1.73, 2.07)
Model 2 [HR (95%CI)]	1 (ref.)	1.24 (1.12, 1.36)		1.58 (1.44, 1.74)
2017 ACC/AHA	Normal	Elevated	Stage 1	Stage 2
No. of AF cases	941	342	382	1226
No. of participants	6249	1562	2057	5047
Person-years	142,742	33,183	44,666	98,426
Incidence rate (per 1000 PY)	6.6	10.3	8.6	12.5
Model 1 [HR (95%CI)]	1 (ref.)	1.36 (1.20, 1.54)	1.24 (1.10, 1.40)	1.90 (1.73, 2.07)
Model 2 [HR (95%CI)]	1 (ref.)	1.26 (1.11, 1.43)	1.21 (1.07, 1.37)	1.58 (1.44, 1.74)

Model 1: Adjusted for age, sex, and race

Model 2: Adjusted for age, sex, race, height, education, field center, body mass index, smoking, drinking status, diabetes, heart failure, coronary heart disease, and stroke

*JNC 7* 7th Joint National Committee, *ACC/ AHA* American College of Cardiology/American Heart Association, *PY* person-years

### Population attributable fraction of the JNC7 and 2017 AHA/ACC guidelines

Using the JNC 7 guidelines, the PAFs were 4% (95% CI 2, 6) for prehypertension and 13% (95% CI 11, 16) for hypertension. In contrast, using categories defined in the 2017 ACC/AHA guidelines, the PAFs were 2% (95% CI 1, 3), 2% (95% CI 1, 3), and 13% (95% CI 11, 16) for elevated, stage 1 and stage 2 hypertension, respectively (Table 5).

**Table 5:** Rate Ratios and Population Attributable Factor of Atrial Fibrillation by Blood Pressure Categories According to JNC 7 and 2017 ACC/AHA Guidelines, ARIC 1987–2015

JNC 7	Normal	Prehypertension		Hypertension
Prevalence, %	41.9	24.3		33.8
RR (95%CI)^a^	1 (ref.)	1.20 (1.09,1.33)		1.46 (1.33, 1.60)
PAF % (95% CI)		4 (2, 6)		13 (11, 16)
2017 ACC/AHA	Normal	Elevated	Stage 1	Stage 2
Prevalence, %	41.9	10.5	13.8	33.8
RR (95% CI)^a^	1 (ref.)	1.23 (1.09, 1.40)	1.18 (1.05, 1.33)	1.46 (1.33, 1.60)
PAF % (95% CI)		2 (1, 3)	2 (1, 3)	13 (11, 16)

^a^ Adjusted for age, sex, race, height, education, field center, body mass index, smoking, drinking, diabetes, heart failure, coronary heart disease, and stroke

*JNC 7* 7th Joint National Committee, *ACC/ AHA* American College of Cardiology/American Heart Association, *RR* Rate Ratios, and *PAF* Population Attributable Factor

When hypertension was considered as a dichotomous variable, the prevalence of hypertension was 34% using the JNC 7 definition and 48% with the 2017 ACC/AHA definition. The PAF for hypertension was 11% (95% CI 8, 13) and 13% (95% CI 9, 16) under the old and new guidelines respectively (Table 6).

**Table 6:** Rate Ratios and Population Attributable Factor of Atrial Fibrillation by Hypertension Definition According to JNC 7 and 2017 ACC/AHA Guidelines, ARIC 1987–2015

JNC 7	No hypertension	Hypertension
Prevalence, %	66.2	33.8
RR (95%CI)^a^	1 (ref.)	1.34 (1.24, 1.45)
PAF % (95% CI)		11 (8, 13)
2017 ACC/AHA	No hypertension	Hypertension
Prevalence, %	52.4	47.6
RR (95%CI)^a^	1 (ref.)	1.29 (1.19, 1.40)
PAF % (95% CI)		13 (9, 16)

^a^ Adjusted for age, sex, race, height, education, field center, body mass index, smoking, drinking, diabetes, heart failure, coronary heart disease, and stroke

*JNC 7* 7th Joint National Committee, *ACC/ AHA* American College of Cardiology/American Heart Association, *RR* Rate Ratios, and *PAF* Population Attributable Factor

## Discussion

In this analysis of a large community-based cohort, we found that blood pressure categories are linearly associated with incidence of AF using both the JNC7 and the 2017 ACC/AHA definitions. However, despite a 40% increase in the prevalence of hypertension using the new 2017 ACC/AHA definition compared to JNC7 (from 33 to 48%), the population impact of the new definition (as characterized by PAF) was limited, with the PAF for hypertension increasing from 11% using the JNC7 definition to 13% with the new 2017 ACC/AHA definition. These results suggest that the extended definition of hypertension based in the guidelines may have a limited additional impact on the prevention of AF.

Hypertension is very common among individuals with AF, both conditions frequently coexisting. It has the largest population attributable fraction for AF incidence and is an important focus in the management and prognosis of AF [3–5]. Studies have also shown that once hypertension occurs, an individual is predisposed to developing AF even if the blood pressure improves in later years [3]. Thus, understanding the risk of AF in association with hypertension is crucial in preventing AF, reducing AF incidence rates, and subsequently preventing strokes. Several guidelines have been released over the years to identify individuals with hypertension based on an increased risk of adverse outcomes and prevent its deleterious consequences by keeping blood pressure at optimal levels. The most recent hypertension guideline, released in the fall of 2017 by the ACC/AHA, refined the guidelines released by JNC7 and JNC8 by lowering the threshold to define hypertension. Consequently, more individuals are diagnosed with hypertension under the new guidelines. The rationale for this change is based on the observed increased risk of cardiovascular disease among individuals in the JNC7 prehypertensive category and results from the SPRINT trial, showing cardiovascular benefit in the treatment of blood pressure, targeting a SBP of < 120 mmHg [16]. However, the risk of AF among individuals diagnosed with hypertension using the new guidelines is uncertain. Our results suggest that the risk of AF in participants with stage 1 hypertension according to the 2017 guidelines (part of the prehypertension category using the JNC7 definition) is only moderately increased compared to normotensive individuals.

We estimated that the PAF for AF from hypertension using the new guidelines barely increased compared to hypertension defined using the JNC7 definition. A prior analysis of the ARIC population showed that borderline blood pressure levels (SBP 120–139 mmHg or DBP 80–89) explained an additional 3% of AF cases. In that prior ARIC analysis, PAF from hypertension was 22% of incident AF and this number increased to 24% adding borderline levels of blood pressure, [5] consistent with our new results. Discrepancies in the overall PAF from hypertension could be explained by the longer follow-up in the new analysis and the more careful adjustment for potential confounders.

Our findings have two major clinical implications. First, we show that even small elevations in BP beyond what is considered normal using the new hypertension definition are associated with increased risk of AF. Pending results from randomized trials testing intensive blood pressure control for AF prevention, our findings suggest that at the individual level, greater blood pressure reduction may reduce the risk of AF. Second, our findings suggest that, at the population level, prevention and treatment of hypertension among those with moderately elevated blood pressure may be only of marginal benefit for AF prevention. These findings can be useful to inform public health policies and the allocation of scarce resources for prevention. Future randomized trials of blood pressure control using AF as a prespecified endpoint are needed to define the impact of blood pressure treatment and control.

### Strengths and limitations

Our study had important strengths. First, we had a large sample size and long follow-up. The study participants were from four geographically diverse communities and the final sample size included 14,915 individuals, with 2891 AF events, providing enough events in each category. Additionally, our study had extensive information on other risk factors for AF, allowing us to adjust for potential confounders. However, there were several limitations in our study. First, though we adjusted for major potential confounders, other common causes of hypertension and AF may have biased the results. Secondly, the study did not differentiate between AF subtypes such as paroxysmal, persistent, or permanent AF. Thirdly, some cases of AF may have been missed due to the method of AF ascertainment, which relies on hospital discharge codes, ECGs conducted at study visits, and death certificates. Thus, paroxysmal and asymptomatic AF cases would have been less likely to be identified. Finally, though our results can be generalizable to other populations without repeated measures of blood pressure. we only considered baseline blood pressure measurement and, therefore, our analysis does not evaluate the impact of trajectories of blood pressure over time, which are known to impact AF risk [17].

## Conclusions

In conclusion, our study showed that hypertension defined using the 2017 AAC/AHA guidelines only led to slight increases in PAF values. These results indicate that controlling blood pressure using the targets included in the new hypertension guidelines may have a limited impact in the burden of AF in the population. Additional studies are needed to confirm these observations.

## Supplementary information

**Additional file 1: Table S1.** Hazard Ratios (95% Confidence Intervals) of Atrial Fibrillation by Hypertension Definitions Stratified by Race and Sex, ARIC 1987–2015

## Data Availability

The datasets generated and/or analyzed during the current study are not publicly available due to privacy restrictions but are available from the ARIC Coordinating Center on reasonable request.

## Abbreviations

ACC/AHA: American College of Cardiology/American Heart Association
AF: Atrial fibrillation
CI: Confidence intervals
DBP: Diastolic blood pressure
HR: Hazard ratio
JNC 7: 7th joint National Committee
PAF: Population attributable factor
RR: Rate ratios
SBP: Systolic blood pressure
