# Supplementary material for: Association and impact of hypertension defined using the 2017 AHA/ACC guidelines on the risk of atrial fibrillation in The Atherosclerosis Risk in Communities study
Source: BMC Cardiovasc Disord. 2019 Nov 26;19:262. doi: 10.1186/s12872-019-1259-0 (PMC6878648; doi:10.1186/s12872-019-1259-0)
Supplement: Supplementary file 1 — Additional file 1: Table S1. Hazard Ratios (95% Confidence Intervals) of Atrial Fibrillation by Hypertension Definitions Stratified by Race and Sex, ARIC 1987–2015 [file 12872_2019_1259_MOESM1_ESM.docx]

**Additional file 1: Table S1. Hazard Ratios (95% Confidence Intervals) of Atrial Fibrillation by Hypertension Definitions Stratified by Race and Sex, ARIC 1987-2015**

| **JNC 7** | **Hypertension** |
| --- | --- |
| **Women** | 1.53 (1.36, 1.73) |
| **Men** | 1.35 (1.21, 1.51) |
| **p-value for interaction** | 0.20 |
| **Whites** | 1.40 (1.28, 1.54) |
| **Blacks** | 1.53 (1.27, 1.85) |
| **p-value for interaction** | 0.77 |
|  |  |
| **2017 ACC/AHA** | **Hypertension** |
| **Women** | 1.55 (1.38, 1.75) |
| **Men** | 1.23 (1.10, 1.37) |
| **p-value for interaction** | 0.01 |
| **Whites** | 1.37 (1.25, 1.49) |
| **Blacks** | 1.32 (1.07, 1.63) |
| **p-value for interaction** | 0.55 |

Adjusted Age, sex, race, height, education, field center, body mass index, smoking, drinking status, diabetes, heart failure, coronary heart disease, and stroke. Hypertension according to JNC7 defined as systolic blood pressure ≥140 mmHg or diastolic blood pressure ≥90 mmHg or use of antihypertensive medication. Hypertension according to 2017 ACC/AHA defined as systolic blood pressure ≥130 mmHg or diastolic blood pressure ≥80 mmHg or use of antihypertensive medication.
